# Effectiveness of 10-valent pneumococcal conjugate vaccine against radiologically confirmed pneumonia and invasive pneumococcal disease among young children in Mozambique

**DOI:** 10.1016/j.vaccine.2026.128237

**Published:** 2026-01-19

**Authors:** Betuel Sigaúque, Leocádia Vilanculos, Alberto Chaúque, Benild Moiane, Hélio Mucavele, Sérgio Massora, Sozinho Acácio, Charfudin Sacoor, Llorenç Quintó, Benigna Matsinhe, Fabiana Pimenta, Cynthia G. Whitney, Fernanda C. Lessa, Maria da Glória Carvalho, Jennifer R. Verani

**Affiliations:** a Centro de Investigação Em Saúde Da Manhica(CISM), Manhica, Mozambique; b John Snow (JSI) Research & Training Institute, Inc., Mozambique; c Instituto Nacional de Saúde (INS), Ministério da saúde, Mozambique; d Barcelona Institute for Global Health (ISGlobal), Barcelona, Spain; e National Directorate of Public Health, Ministry of Health, Mozambique; f Division of Bacterial Diseases, Centers for Disease Control and Prevention, Atlanta, United States of America; g International Infection Control Branch, Division of Healthcare Quality Promotion, Centers for Disease Control and Prevention, Atlanta, United States

**Keywords:** PCV10, Pneumonia, Invasive pneumococcal disease, Vaccine effectiveness

## Abstract

**Background:**

Mozambique introduced the 10-valent pneumococcal conjugate vaccine (PCV10) in 2013. We aimed to assess PCV10 effectiveness against radiologically confirmed pneumonia (RCP) and invasive pneumococcal disease (IPD) among young children in southern Mozambique.

**Methods:**

We conducted a case-control vaccine effectiveness (VE) study at 2 sites (Mavalane and Manhiça) from January 2014 to December 2016. Children age-eligible to have received PCV10 admitted with pneumonia underwent chest radiograph with standardized interpretation as per the World Health Organization and had nasopharyngeal swabs collected and cultured. IPD cases were recruited in Manhiça only, leveraging existing surveillance; IPD was defined as pneumococcus detected through blood culture or cerebrospinal fluid culture and/or PCR with serotyping by Quellung or PCR. We enrolled age-matched community controls (date of birth ±1 month for cases aged <6 months and +/− 2 months for cases aged ≥6 months). VE was estimated using multivariate logistic regression with 0 doses as a comparator.

**Results:**

Among 812 enrolled RCP cases, 780 (96.1%) had matched controls. The median age of included cases was 11.6 months and 51.8% were male; 94.4% of cases and 97.0% of controls had ≥1 PCV10 dose. Adjusted VE against RCP was 23.5% (95% confidence interval [95%CI]: −31.9, 55.6) for 2 doses and 47.2% for 3 doses (95% CI: 13.7, 67.7). Adjusted VE of ≥2 doses and exactly 3 doses was 36.8% (95CI%: −11.9, 64.3) and 41.6% (95CI%: −5.7, 67.7), respectively. Models for VE against RCP with PCV10-type carriage yielded negative point estimates with wide confidence intervals. Among 26 enrolled IPD cases, 24 (92.3%) had matched controls (including 8 PCV10-type cases); 91.7% of IPD cases and 100% of controls had ≥1 PCV10 dose. IPD VE models did not converge.

**Conclusion:**

PCV10 offers substantial protection against RCP in young children in a high burden setting, highlighting its importance for reducing child pneumonia globally.

## 1. Introduction

*Streptococcus pneumoniae* (pneumococcus) is a leading cause of bacterial pneumonia and invasive disease in young children, particularly in low- and middle-income countries (LMIC) [1,2]. Pneumococcus is the most frequent etiology implicated in deaths from lower respiratory infections globally [3], and was estimated to have caused more than 200,000 deaths among children under five years old in 2019 [4]. The burden of pneumococcal pneumonia and deaths is highest in sub-Saharan Africa and South Asia [5].

Pneumococcal conjugate vaccines (PCV) are an essential tool for reducing the burden of pneumococcal diseases. Currently, most countries worldwide use PCV in their routine infant immunization programs [6] and the most widely used formulations are 10-valent (GlaxoSmithKline, PCV10) and 13-valent (Pfizer, PCV13). Data from both high-income settings and LMICs have demonstrated high PCV impact [7,8] and effectiveness [9,10] against vaccine-type invasive pneumococcal disease (IPD). Studies have also demonstrated declines in pneumonia hospitalizations following PCV introduction [11,12], although available evidence is disproportionately from high- and upper-middle-income countries. Data on PCV effectiveness against pneumonia using the case-control method, which complements data on vaccine impact [13], are limited [14–17]. Assessing how well PCV protects against pneumonia is challenging methodologically because of the low specificity of the outcome, but it is important from a policy perspective because of the high burden of pneumonia [2]. The World Health Organization (WHO) developed standardized criteria for interpretation of chest radiographs to define ‘primary endpoint pneumonia’, to be used as an endpoint in bacterial vaccine trials [18]; this outcome has also been used by studies evaluating the effectiveness of PCV against pneumonia in real-world settings.

Mozambique introduced PCV10 into its Expanded Program on Immunization in April 2013 using three primary doses at 2, 3, and 4 months (3 + 0 schedule) with no catch-up campaign. We leveraged an established platform for pneumonia and invasive bacterial disease surveillance to assess PCV10 effectiveness against radiologically confirmed pneumonia (RCP) using WHO criteria [18] and against IPD among young children in southern Mozambique.

## 2. Methods

### 2.1. Study setting

From January 2014 to December 2016, we conducted a case-control study to estimate vaccine effectiveness (VE) against RCP and IPD among children age-eligible to have received PCV10 in two sites in southern Mozambique: Manhiça district and Mavalane health catchment areas. Manhiça district is a predominantly rural area where the Manhiça Health Research Centre (CISM) is located and operated a Health and Demographic Surveillance System (HDSS) that covered approximately 200,000 inhabitants in an area of 2380 km^2^ [19,20]. Mavalane is an urban neighborhood of Maputo city, the capital of Mozambique. This neighborhood is located approximately 5.5 km from the city center of Maputo and has estimated 400,000 inhabitants and covers approximately 168.4 km^2^_._ Both Manhiça and Mavalane are high HIV prevalence areas [21].

For the Manhiça site, case enrollment was conducted at Manhiça District Hospital, which is the main public hospital within the district and has approximately 50 beds in the pediatric ward. This hospital serves a network of 15 peripheral health centers. In the Mavalane site, case enrollment was conducted at two hospitals: Mavalane General Hospital and Maputo Central Hospital. Mavalane General Hospital is the main public hospital serving the Mavalane neighborhood with approximately 120 beds in capacity for the pediatric ward. This hospital serves 11 peripheral health centers. Maputo Central Hospital is a national referral and medical teaching hospital in Maputo with approximately 350 beds in the pediatric ward. This hospital is not located within the Mavalane health catchment area, yet was added as an extension to Mavalane to capture participants from the Mavalane catchment area who may have presented directly to this hospital or transferred from Mavalane General Hospital before recruitment.

### 2.2. Study population

The study included children who were residents of the study areas and age-eligible to have received at least 1 dose of PCV10 through the national immunization program at least 2 weeks prior to enrollment (to allow for immune response to vaccination), i.e. born after December 10, 2012, and ≥ 10 weeks old at the time of enrollment.

### 2.3. Identification and enrollment of cases

RCP cases were identified and enrolled at the three study hospitals. In Manhiça District Hospital, RCP cases were identified through ongoing morbidity surveillance that routinely captured clinical and radiologic data on hospitalizations for clinical severe pneumonia [22], defined as cough or difficulty breathing plus tachypnea and chest wall-indrawing or stridor in a calm child [23,24]. A chest X-ray (CXR) was performed for all children admitted to the pediatric ward with the criteria of severe pneumonia. At the Mavalane site hospitals, pneumonia surveillance was established for the study, and clinical pneumonia was defined as a clinical diagnosis of pneumonia or bronchopneumonia in an admitted child. Cases at this site were identified by reviewing clinical files daily in pediatric wards, and CXR was performed within 48 h for all children admitted who met eligibility criteria.

All CXRs were reviewed by two independent readers (two clinicians or a clinician and a radiologist) trained following the World Health Organization (WHO) standardized interpretation of pediatric chest radiographs [18]. Cases meeting the WHO criteria for endpoint pneumonia were included in the study as RCP.

IPD cases were enrolled at the Manhiça site only, where surveillance for invasive bacterial disease had been ongoing since 2004 [22]. All hospitalized children aged <24 months or with temperature ≥ 39 °C or clinical signs of infection (e.g., clinical instability and/or focal signs of infection) had whole blood collected for culture [25]. Children with any of the following criteria had cerebrospinal fluid (CSF) collected for culture and polymerase chain reaction (PCR) testing: neurological alterations of any level with normal previous neurological status, meningeal signs (neck stiffness, positive Kerning or Brudzinski signs), tense fontanelle (if age < 1 year old), altered level of consciousness (Glasgow Coma Schedule <15), or two or more episodes of seizures, without a clinical history of epilepsy or suspected typical febrile seizures [26,27]. IPD was defined as *S. pneumoniae* detected in blood or CSF (see laboratory methods below). Vaccine-type IPD cases were those caused by a PCV10 serotype (1, 4, 5, 6B, 7F, 9 V, 14, 18C, 19F and 23F).

### 2.4. Identification and enrollment of controls

We enrolled age-matched community controls for RCP and IPD cases. For cases aged <6 months, eligible controls had a date of birth ±1 month of the case, and for cases aged ≥6 months, controls had a date of birth +/− 2 months. Controls were recruited as soon as possible after enrollment of the case. We aimed to enroll 4 controls per case.

In the Manhiça site, a list of eight randomly selected potential community controls was generated from the Manhiça Health and Demographic Surveillance System (HDSS) database for each case within one week of case recruitment. The selection of potential controls started with age-eligible children who resided closest to the case (excluding children from the same household) within 500 m; if fewer than four controls were recruited within this area, the perimeter was increased by another 500 m and so on until the desired number of controls was reached. Field workers visited potential control households to invite them to participate in the study in order of the geographically ordered list until four controls per case were enrolled. In the Mavalane site, a modified random walk door-to-door starting from the case’s house was used to locate potential controls [28]. In both sites, field teams returned to the house of potential control at least 3 times if initially no one was home. Children who were previously enrolled as cases or controls in this study were not eligible.

### 2.5. Data and sample collection

A standardized questionnaire was used to collect demographic, household and socioeconomic data for cases and controls via interview of the parent or guardian. Cases additionally had clinical information abstracted from the medical record. For both cases and controls, the parent or guardian was asked about receipt of any vaccine beyond birth doses; for those reporting receipt of any vaccine, vaccination data were collected from the immunization card. If the card was not available, vaccination status was obtained from the health records facility where the child was reportedly vaccinated.

Enrolled pneumonia cases had a single nasopharyngeal swab (NP) collected as soon as possible after admission within 48 h to assess pneumococcal colonization. Swabs were placed in cryotubes containing 1.0 ml skim milk, tryptone, glucose, glycerol (STGG) media, stored at 2–8 °C for up to 4 h, then vortexed for 10–20 s and frozen at −80 °C until processing [29].

### 2.6. Laboratory methods

For NP swab processing, 200 μl of inoculated STGG media was transferred to 5.0 ml of Todd Hewitt broth containing 0.5% yeast extract plus 1 ml of rabbit serum and was incubated at 37 °C with 5% CO_2_ for 6 h. After incubation, 10 μl of cultured broth was inoculated on 5% sheep blood agar plate and incubated for 18–24 h at 37 °C with 5% CO_2_ [30]. Alpha-hemolytic colonies with typical pneumococcal morphology were isolated and identified by optochin susceptibility and bile solubility tests. Pneumococcal serotype was determined for all pneumococcal isolates by the Quellung reaction. Isolates non-typeable by Quellung underwent quantitative PCR (qPCR) for pneumococcal *lyt*A gene detection [31] *lyt*A positive isolates were submitted for serotyping by conventional multiplex PCR (cmPCR) [30]. All culture-negative NP swabs underwent qPCR targeting *lytA* for pneumococcal DNA detection. DNA extracts were obtained from 400uL of STGG NP-swab following the steps described in [32] for MagNa Pure Compact instrument, external lysis protocol, according to manufacturer instructions. All *lytA*-positive NP swab DNAs were serotyped by multiplex qPCR using Quanta Biosciences PerfeCTa Multiplex qPCR ToughMix, Low ROX for 21 assays encompassing 37 serotypes [33].

For children meeting criteria for blood culture, 1–3 ml of whole blood was inoculated into a pediatric blood culture bottle (Peds Plus^®^, Becton-Dickinson, Franklin Lakes, NJ) and incubated in an automated culture system (Becton-Dickinson BACTEC^®^ FX) up to 5 days, following manufacturer instructions. Positive blood culture underwent standard bacterial isolation and identification protocols [34].

CSF samples from children with suspected meningitis underwent Gram stain and culture, also following bacterial isolation and identification standard protocols [34]. All CSF samples were additionally tested by qPCR for *lytA* gene detection and *lytA*-positive samples were serotyped by multiplex qPCR using DNA extraction and qPCR reactions protocols as described above for pneumococcal detection and serotyping in NP swab samples. All pneumococcal isolates from blood cultures and CSF samples were serotyped by Quellung reaction.

### 2.7. Sample size estimation

Assuming a power of 80%, a two sided significance level of 5%, PCV10 coverage of 80% among controls, a control: case ratio of 4:1, and an effectiveness of 25% against radiologically confirmed pneumonia, the minimum sample size of radiologically confirmed pneumonia cases needed was 704.

For IPD, we aimed to recruit all eligible cases during the study period. Assuming a power of 80%, a confidence interval of 95%, PCV10 coverage of 80% among controls, a control: case ratio of 4:1, and an effectiveness of 90%, the minimum number of cases for PCV10-type IPD needed was 12.

### 2.8. Statistical analysis

Only cases of RCP and IPD and their matched controls were included in the analysis. Cases and controls with no matched pairs or missing vaccination history were excluded. Children who received doses of PCV13 (which was not included in the national immunization program but was available in neighboring South Africa) were not included in the analysis. Vaccine doses received at least 14 days before enrollment for cases were considered valid; for controls, doses received at least 14 days before their case’s enrollment date were valid. Children with an immunization card with no documented PCV10 valid doses were considered unvaccinated. Children with a verbal report of no vaccine receipt beyond birth doses were also considered unvaccinated. To compare characteristics of cases and controls, we used the exact McNemar and Stuart Maxwell test for categorical variables and the paired *t*-test for continuous variables.

We estimated VE against RCP; as an exploratory secondary objective, we also estimated VE against RCP plus PCV10-type colonization, hypothesizing that the subset of RCP cases with PCV10-type colonization would be relatively more enriched for vaccine-preventable pneumococcal pneumonia. For IPD, we aimed to estimate VE against all IPD and PCV10-type IPD. Conditional logistic regression was used to estimate VE by comparing PCV vaccination among cases and controls using the formula VE = (1-odds ratio for PCV vaccination)*100%. For each outcome, we ran four models with different classifications of PCV exposure: [1] number of PCV10 doses (i.e. 1, 2, or 3 doses) vs 0 doses, [2] ≥1 doses vs 0 doses, [3] ≥2 doses vs 0 doses, and [4] 3 doses vs 0 doses. For the models of VE of ≥2 doses and 3 doses, children not age-eligible for that number of PCV10 doses were excluded. Potential confounders were evaluated by including them one by one in a basic VE model (outcome case/control status and exposure PCV vaccination) and assessing their impact on VE estimates; factors that changed the odds ratio by >10% were included in adjusted VE models. We also examined for collinearity and effect modification among variables included in the final VE models. Statistical analysis was performed using the R software program version 4.3.1.

### 2.9. Ethical considerations

We obtained written informed consent from parents or guardians of cases and controls prior to participation in the study. The study was approved by the CISM scientific committee and the National Committee for Bioethics and Health in Mozambique. This activity was reviewed by the CDC, deemed not research, and was conducted consistent with applicable federal law and CDC policy.^2^

## 3. Results

**Fig. 1.**
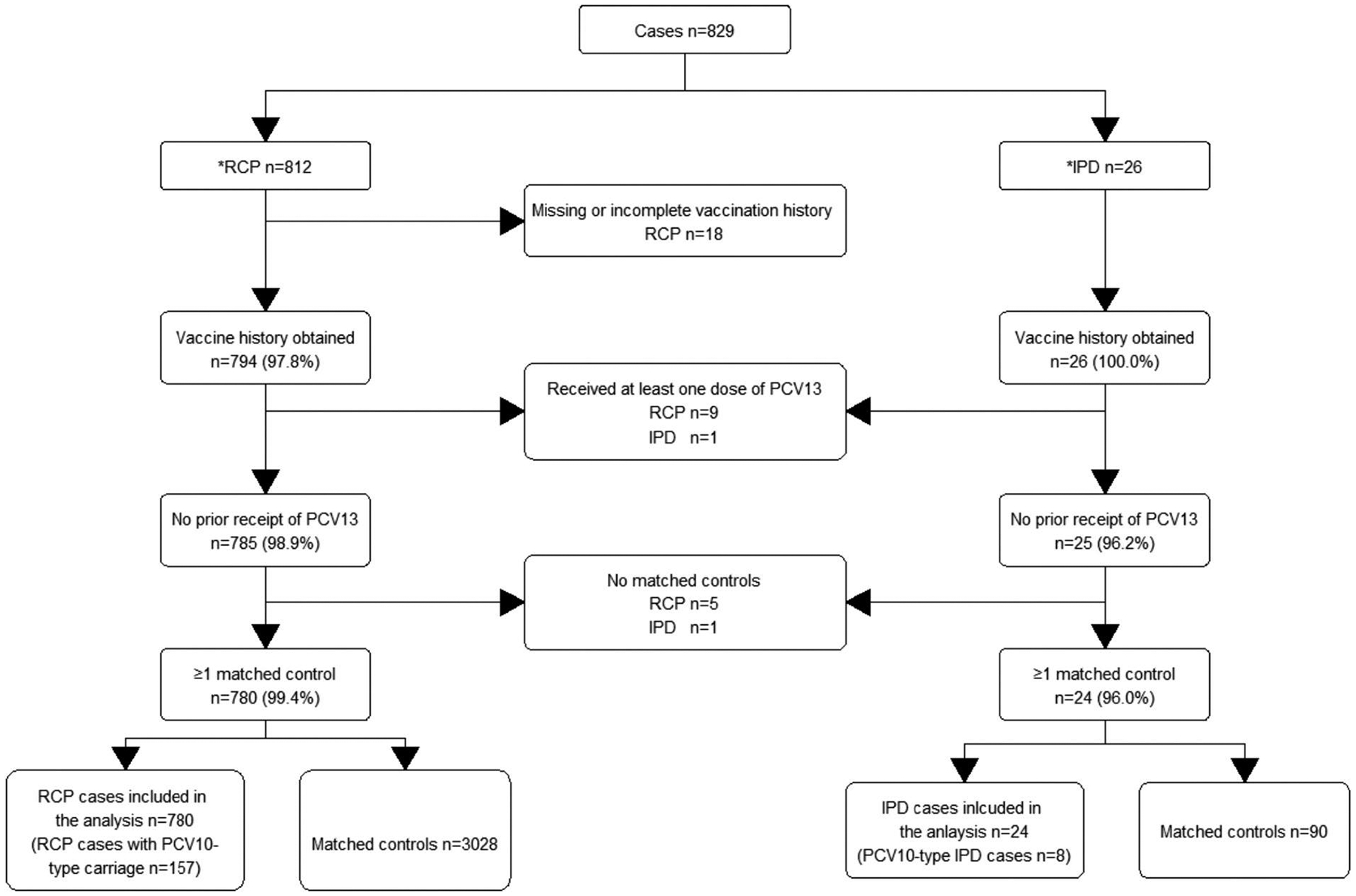
Enrollment of radiologically confirmed pneumonia (RCP) and invasive pneumococcal disease (IPD) cases and matched controls in Mozambique * 9 individual cases meeting criteria for both RCP and IPD included in both branches of the flow chart **Note:** A total of 181 controls of cases included in the final analysis were excluded, including 82 due to lack of vaccination and 25 who had received the PCV13 vaccine.

We recruited a total of 812 RCP cases, from whom 780 (96.1%) had matched controls and were included in the analysis (Fig. 1). The median age of RCP cases with matched controls was 11.6 months, and 51.8% were male. RCP cases and their matched controls had similar demographic characteristics, although low birth weight, day care attendance and having an HIV-infected mother were more commonly observed in cases (Table 1). Among RCP cases included in the analysis, 85.5% had received two or more valid doses of PCV and 5.6% had zero doses; among matched controls, 90.8% had two or more valid doses and 3.0% had zero doses.

For VE estimation, low birth weight and receipt of the first polio dose (given at birth or given within the first month of life for low-birth-weight babies) met criteria for inclusion as a covariate in adjusted models. Adjusted VE against RCP estimated by the model with PCV classified as number of doses (1, 2, or 3 doses vs. 0 doses) was 23.5% (95% confidence interval [95%CI]: −31.9, 55.6) for 2 doses and 47.2% (95%CI: 13.7, 67.7) for 3 doses (Table 2). Models of the VE of ≥2 doses and exactly 3 doses (vs. 0 doses) yielded adjusted estimates of 36.8% (95CI%: −11.9, 64.3) and 41.6% (95CI%: −5.7, 67.7), respectively. Adjusted estimates of VE against RCP plus PCV10-type colonization had negative point estimates with wide confidence intervals across all models (Table 2).

A total of 26 IPD cases were recruited from whom 24 (92.3%) had matched controls and were included in the analysis (Fig. 1), including 8 PCV10-type cases. The median age of IPD cases was 10.6 months and 58.3% were male. IPD cases had similar demographic characteristics as their matched controls, although being born to an HIV-infected mother was more common in cases (Table 1). Among IPD cases included in the analysis, 79.2% had received two or more doses and 8.3% had not received any doses; among the matched controls, 92.2% had two or more doses and none was unvaccinated. Models did not converge to allow estimation of VE against IPD or PCV10-type IPD.

## 4. Discussion

This study provides evidence of PCV10 effectiveness in preventing WHO-defined RCP in young children in Mozambique, contributing to a very limited evidence base of real-world PCV protection against pneumonia in regions of the world with the highest burden of disease. These data suggest that a full schedule of PCV in the routine infant immunization program can reduce the risk of RCP by nearly 50%. Although we were not able to estimate VE against IPD due to limited statistical power, the small number of IPD cases enrolled in this setting, with a previously demonstrated high burden of pneumococcal disease, is consistent with a robust PCV impact [7,8]. Because pneumonia remains a leading cause of death for children in LMIC [2], these data highlight the importance of PCV for achieving Sustainable Development Goal targets for reducing child mortality [35].

The WHO approach for defining ‘endpoint pneumonia’ based on chest radiograph was developed as a standardized pneumonia outcome and was used for seminal PCV clinical trials, which reported PCV efficacy against RCP of 23–37% [36–38]. However, observational data on the effectiveness of PCV against RCP in LMICs which have rolled out PCV in routine infant immunization programs are limited. A matched case-control study of the effectiveness of PCV7 /PCV13 against radiologically confirmed presumed bacterial pneumonia (using WHO radiologic criteria) in South Africa conducted from 2009 to 2012 estimated that the VE of an age-appropriate number of PCV doses among children eligible for at least two doses was 39.2% (95%CI: 8.4, 59.6) using hospital controls and 52.7% (95%CI: 25.7, 69.9) using community controls [39]. A study in the Gambia examining the effectiveness of PCV13 against WHO-defined RCP using matched community controls from 2011 to 2014 reported an adjusted odds ratio of 0.78 (95:CI 0.47, 1.30) for two or three doses, which translates to a VE of 22% with a confidence interval that spans zero [40]. A matched case-control study of PCV10 effectiveness against WHO-defined RCP carried out in 2015 to 2017 in Bangladesh reported a VE of 21.4% (95%CI: −0.2, 38.4) for two or more doses using clinic controls and 7.6% (95%CI: −22.2, 30.0) using community controls; when restricting to children aged 3–11 months and using clinic controls, the VE of 3 doses was 47.3% (95%CI: 10.5, 69.0) [14]. Case control VE studies are susceptible to bias and confounding, particularly for non-specific outcomes such as pneumonia [13], and the authors of the Bangladesh study posited that residual bias may have contributed to the lack of significant protection when using community controls. Nonetheless, our estimates using a similar design as these three studies provide additional real-world evidence of the role of PCV in reducing pneumonia among young children globally.

Measuring PCV VE against pneumonia is challenging because of the low specificity of the outcome, since pneumonia can be caused by many non-pneumococcal pathogens and by non-vaccine pneumococcal serotypes. In addition to the WHO ‘end point’ pneumonia, other strategies have been used to craft a relatively more specific pneumonia outcome to assess PCV VE. The Bangladesh VE study mentioned above included a component examining sonographic-confirmed pneumonia that reported an adjusted VE of ≥ 2 doses of PCV10 using clinic controls was 15.8% for consolidations ≥ 1 cm and 41.1% for consolidations ≥ 2 cm on ultrasound [15]. Hypoxemic pneumonia has been used as an outcome for PCV observational studies, including assessments of PCV impact on disease burden in the Gambia [40] and Malawi [41]. Hypoxemic pneumonia was also the outcome for studies using a test-negative design to estimate PCV13 VE in Laos [42] and Papua New Guinea [43], comparing the vaccination status of pneumonia cases with hypoxemia to that of pneumonia cases without hypoxemia, yielding VE estimates of 35% and 28.7%, respectively. For our study, we examined pneumococcal colonization among RCP cases and hypothesized that RCP plus PCV10-type carriage might be a more specific outcome for the vaccine-preventable portion of RCP and thus yield a higher VE estimate. A prior analysis of data from this setting found that vaccine-type carriage was more common among children with RCP compared with children in the community without RCP (adjusted OR 1.4, 95CI: 1.10–1.78) [32]. However, pneumococcal carriage in this setting is common among children aged <5 years, both with and without pneumonia and restricting to RCP cases with PCV10-type carriage reduced case counts and statistical power. Our examination of VE against this outcome did not yield meaningful results.

Although we intended to assess VE against IPD, we were not able to due to a small number of IPD cases and high PCV coverage. We enrolled fewer PCV10-type IPD cases (*n* = 8) than the target number based on sample size calculations (*n* = 12); furthermore, there were no unvaccinated IPD controls. Prior to PCV introduction in Mozambique, a high burden of IPD was observed in Manhiça, with incidence ranging from 106 to 531 per 100,000 child–years at risk [27,44]; In 2016, within 3 years following PCV introduction, the incidence had declined to 41 per 100,000 [45]. Data from case control VE studies in Southern Africa using different PCV formulations (PCV7 and PCV13) have reported VE against vaccine-type IPD between 74 and 92% [46–48]. Although we have not contributed to that evidence base, the small number of identified cases in this setting represents the success of the PCV program in reducing the burden of IPD.

Beyond having inadequate power to estimate VE against IPD, our study had additional limitations. Several of the models estimating VE against RCP had wide, non-significant confidence intervals, reflecting limited statistical power. PCV coverage was higher than the anticipated 80%, which reflects the success of the immunization program in rolling out PCV10, but negatively impacted statistical power of this study. Although both sites enrolled RCP cases based on standardized interpretation of radiographs, the case definition used to identify pneumonia cases differed across the two sites; in Manhiça, it was based on signs and symptoms, while in Mavalane it was based on clinician diagnosis. The methods for identifying potential controls also differed across sites, drawing from the HDSS database in Manhiça and using a modified random walk procedure in Mavalane; it is possible that the modified random walk could be more susceptible to bias since it does not draw from a population-based list of potentially eligible children. As with all case-control VE studies, there is potential for confounding particularly when vaccine coverage is high; although we included in the VE model covariates that met pre-set criteria for confounders, it is possible that unmeasured or inadequately captured confounders could have biased VE estimates. The exclusion of some participants due to a lack of matched control or no documented vaccination history could have introduced bias.

Despite these limitations, this study provides additional evidence of the substantial protective effect of PCV10 against pneumonia in young children in a high-burden setting. Mozambique subsequently changed the PCV product (switched from PCV10 to PCV13 in 2018) and schedule (switched to two primary doses plus a booster dose in 2019), with the aim of optimizing the impact of the PCV program. This study demonstrates the value of PCV in the country’s routine infant immunization program and highlights its role in reducing childhood pneumonia globally.

## Figures and Tables

**Table 1:** Characteristics of enrolled cases of radiologically confirmed pneumonia (RCP), invasive pneumococcal disease (IPD), and their matched controls in Mozambique.

	RCP cases	RCP controls	*p*-value	IPD cases	IPD controls	p-value
	(*n* = 780)	(*n* = 3028)		(*n* = 24)	(*n* = 90)	
	n (%)	n (%)		n (%)	n (%)	
Median age in months at recruitment (range)	11.6 (7.0)	11.6 (7.3)	0.5	11 (6.5)	10.6 (6.7)	0.5
Male sex	404 (51.8)	1.571 (51.9)	0.7	14 (58.3)	38.0 (42.2)	**0.010**
Low birth weight*	84 (12.1)	242 (8.3)	<**0.001**	2 (11.1)	16.0 (18.4)	0.6
Preterm^†^	55 (21.3)	251 (17.5)	0.3	6 (42.9)	19.0 (26.0)	0.6
Mother HIV positive	226 (30.4)	643 (22.6)	<**0.001**	16 (72.7)	29.0 (32.6)	<**0.001**
Day care attendance	23 (2.9)	48 (1.6)	<**0.001**	0 (0.0)	1.0 (1.1)	>0.9
Number of household members	<**0.001**			0.064		
≤2	12 (1.5)	30 (1.0)		0 (0.0)	0.0 (0.0)	
3–4	178 (23.0)	820 (27.1)		9 (39.1)	23.0 (25.6)	
≥5	585 (75.5)	2.176 (71.9)		14 (60.9)	67.0 (74.4)	
Received birth dose of polio vaccine	707 (93.1)	2759 (92.4)	0.3	23 (95.8)	90 (100)	0.082
Number of valid PCV10 doses^‡^	<**0.001**			0.068		
0	44 (5.6)	90 (3.0)		2 (8.3)	0.0 (0.0)	
1	69 (8.8)	188 (6.2)		3 (12.5)	7.0 (7.8)	
2	82 (10.5)	275 (9.1)		2 (8.3)	13.0 (14.4)	
3	585 (75.0)	2475 (81.7)		17 (70.8)	70 (77.8)	
Presence of household members who smoke	164 (21.0)	489 (16.1)	<**0.001**	5 (21.7)	17 (18.9)	0.8
Number of children <5 sleeping in the same room	<**0.001**			0.3		
Zero	536 (69.4)	2252 (74.4)		16 (69.6)	67 (74.4)	
≥ 1	236 (30.6)	774 (25.6)		7 (30.4)	23 (25.6)	
Number of children <5 living in the same house	<**0.001**			0.2		
<2	351 (45.3)	1566 (51.7)		10 (45.5)	48 (53.3)	
≥ 2	424 (54.7)	1461 (48.3)		12 (54.5)	42 (46.7)	
Number of people sleeping in the same room	**0.002**			0.7		
<2	113 (14.6)	358 (11.8)		4 (17.4)	14 (15.6)	
≥ 2	663 (85.4)	2669 (88.2)		19 (82.6)	76 (84.4)	
Source of fuel used for cooking at home	>0.9			>0.9		
Firewood	137 (17.6)	544 (18.0)		19 (82.6)	73 (81.1)	
Others	641 (82.4)	2477 (82.0)		4 (17.4)	17 (18.9)	
Birth Place	0.6			**0.003**		
Home/street	49 (6.3)	175 (5.8)		0 (0.0)	11 (12.2)	
Hospital	731 (93.7)	2844 (94.2)		23 (100)	79 (87.8)	
Number of rooms in the house	<**0.001**			>0.9		
<2	219 (28.3)	1018 (33.6)		12 (52.2)	48 (53.3)	
≥ 2	555 (71.7)	2008 (66.4)		11 (47.8)	42 (46.7)	
Birth by Cesarean section	88 (11.3)	347 (11.6)	0.9	2 (8.7)	9 (10.3)	0.8

† Preterm: babies born alive before 37 weeks of pregnancy are completed.

‡ PCV10 Vaccine doses received at least 14 days before enrollment for cases were considered valid, for controls, doses received at least 14 days before their case’s enrollment date were considered valid.

* Low birthweight: a baby born weighing less than 2500 g.

**Table 2:** Estimated vaccine effectiveness (VE) of 10-valent pneumococcal conjugate vaccine (PCV10) against radiologically confirmed pneumonia (RCP) in Mozambique.

	Number of contributing strata*	Crude VE (95% confidence interval)	Adjusted VE^§^ (95% confidence interval)
**Effectiveness against RCP**			
*VE by number of PCV doses (vs 0 doses)*			
1 dose	577	10.2 (−45.5, 44.5)	−15.6 (−101.0, 33.5)
2 doses	577	35.1 (−3.5, 59.3)	23.5 (−31.9, 55.6)
3 doses	577	57.3 (36.2, 71.4)	47.2 (13.7, 67.7)
*VE of one or more PCV doses (vs 0 doses)*			
≥1 dose	140	46.5 (21.4, 63.6)	29.7 (−12.2, 55.9)
*VE of two or more PCV doses (vs 0 doses)* ^†^			
≥2 doses	100	53.2 (26.9, 70.1)	36.8 (−11.9, 64.3)
*VE of three PCV doses (vs 0 doses)* ^‡^			
3 doses	91	56.3 (30.9, 72.4)	41.6 (−5.7, 67.7)
**Effectiveness against RCP plus PCV10-type carriage**			
*VE by number of PCV doses (vs 0 doses)*			
1 dose	106	−37.2 (−394.5, 61.9)	−86.1 (−715.3, 57.5)
2 doses	106	−20.6 (−350.8, 67.7)	−54.0 (−613.5, 66.8)
3 doses	106	5.5 (−216.9, 71.8)	−16.0 (−385.5, 72.3)
*VE of one or more PCV doses (vs 0 doses)*			
≥1 dose	18	−10.8 (−253.3, 65.3)	−45.1 (−462.5, 62.6)
*VE of two or more PCV doses (vs 0 doses)* ^†^			
≥2 doses	9	−37.5 (−600.2, 73.0)	−162.8 (−2343.1, 71.7)
*VE of three PCV doses (vs 0 doses)* ^‡^			
3 doses	9	−52.3 (−669.7, 69.8)	−185.8 (−2536.4, 69.0)

† Excludes participants with exactly 1 dose.

‡ Excludes participants with exactly 1 or 2 doses.

§ Included low birth weight and receipt of the first polio dose.

* Strata with discordance between cases and at least one matched control for PCV vaccination status.

## Data Availability

Data will be made available on request.

## Footnotes

2 See e.g., 45C.F.R. part 46, 21C.F.R. part 56; 42 U.S.C. §241(d); 5 U.S.C. §552a; 44 U.S.C. §3501 et seq.
